# Association between oral hygiene knowledge and practices among older dental patients attending private dental clinics in Addis Ababa, Ethiopia

**DOI:** 10.1038/s41405-024-00243-2

**Published:** 2024-07-16

**Authors:** Yemisrach Mulatu, Mohammed Mehdi, Yeshewas Abaynew

**Affiliations:** 1 Sitamin Medium Dental Clinic, Addis Ababa, Ethiopia; 2 Biochemistry Department, College of Health Sciences, Addis Ababa University, Addis Ababa, Ethiopia; 3 School of Public Health, College of Medicine and Health Sciences, Wollo University, Dessie, Ethiopia

**Keywords:** Oral diseases, Dentistry

## Abstract

**Background:**

Ensuring optimal oral hygiene is crucial for preserving the well-being of hard and soft tissues within the oral system. A lack of proper oral hygiene can have a detrimental impact on an individual’s health, leading to conditions such as caries and periodontitis. Therefore, this study investigated the association between the knowledge and practices of older patients in oral hygiene in Addis Ababa, Ethiopia.

**Methods:**

A facility-based cross-sectional study was carried out on 111 older patients who presented at purposively selected dental clinics in Addis Ababa. A convenience sampling method was used to recruit study participants. The data were collected using a pretested structured questionnaire. The questionnaire included information related to the patient’s knowledge and practices related to oral hygiene. The data were analyzed using SPSS version 23. Descriptive statistics were used to analyze the data. Multivariate logistic regression was performed to investigate the associations between independent and dependent variables. Adjusted odds ratios with 95% confidence intervals (95% CIs) were estimated, and variables with a *p* value < 0.05 in the multivariate analysis were considered significant.

**Results:**

The median age of the study participants was 70.31 years (65–100). Inadequate oral hygiene knowledge was found in 53.15% of the participants, while more than three-quarters (83.78%) of the older participants practiced poor oral hygiene. Older patients with good oral hygiene knowledge were 7.6 times more likely to practice good oral hygiene practices (AOR, 7.6; 95% CI (2.05–27.9)).

**Conclusions:**

Poor oral health is significantly associated with various health problems, particularly in older people. This study examined the relationship between oral health knowledge and practices in older dental patients and revealed insufficient knowledge and practices among participants. The results showed a notable link between oral health knowledge and practices in this demographic group, highlighting the need for support to improve oral health status. Organizations should increase awareness among older patients to improve their oral health status.

## Introduction

Good oral hygiene practices involve maintaining oral cavity health through active hygiene practices that involve keeping the mouth, teeth, and gums clean. Good oral hygiene through proper practice prevents the loss of teeth due to caries, periodontal disease, bad breath, or the staining of teeth from food and drinks [1, 2]. Common signs and symptoms of poor oral health include poor breathing, accumulation of plaque and calculus, gum bleeding, swollen gums, dry mouth, plaque build-up on the tongue, tooth decay, gum, and periodontal disease [3–5].

The health and longevity of older people are closely related to their oral health. Neglecting proper dental hygiene can have serious health implications, especially when compounded by preexisting conditions such as diabetes, limited daily activities, and access to regular dental care [6–9].

Studies have shown that poor oral health is a growing problem in older adults, and as people age, the risk of developing oral diseases also increases. Most older people rely on others for personal care and activities of daily living. This leads to difficulties in consistently performing oral hygiene and results in poor oral hygiene and sequelae [9, 10].

A study conducted in Europe in 2021 among dentate residents in long-term care facilities revealed that four-fifths of the residents had poor oral hygiene [11]. Another study carried out in Calabrian long-term care facilities in southern Italy showed that the older patients involved had poor oral health [12]. A study conducted in the SELGA in older adults showed a very low demand for oral healthcare and demonstrated poor oral hygiene among participants [13].

A previous study showed that approximately 76.3% of older people do not have inadequate oral health knowledge [14]. A study conducted in Canada showed that adults have insufficient knowledge about dental diseases and their prevention methods [14].

A study at the Loma Linda University School of Dentistry and Public Health in California revealed that 56.9% of the participants had poor oral health knowledge. Older age and education level are factors associated with poor oral health knowledge [15]. A study conducted among older people in the Hong Kong community in 2020 indicated that lower education affects oral hygiene knowledge and practices [16].

A 2015 study of older people in Tonga, a division of the West Region of Cameroon, identified a lack of oral health education or motivation as the main cause of poor oral hygiene [17]. A study conducted in Egypt in 2020 revealed inadequate knowledge and practice among the majority of older people studied, resulting in poor oral hygiene and unhealthy dental status [18].

A study by the Macedonian Humanitarian Association, which supports older and disabled people in Addis Ababa, reported that a lack of sufficient knowledge and awareness of proper oral hygiene practices is one of the reasons for poor oral hygiene practices among older people [19].

Few studies have examined the oral health knowledge and practices of older people. In addition, studies have not established a link between oral hygiene knowledge and practices among older dental patients, especially in Ethiopia. Therefore, this study assessed oral hygiene knowledge and practices and investigated the association between oral hygiene knowledge and practices among older dental patients in Addis Ababa. This study will provide much-needed data that can be used to inform policymakers, program planners, and resource allocators for this population.

## Materials and methods

A facility-based cross-sectional study was conducted at the Nash Specialty Dental Clinic, Sitamin Medium Dental Clinic, Dent-Efrata Dental Clinic, and Merit Medium Dental Clinic in Addis Ababa, Ethiopia. All are located in Addis Ababa, the capital of Ethiopia. The choice of clinics rather than hospitals is likely a trade-off between practicality and availability of resources, as clinics cover a wider range of patients suitable for the study. Clinics were selected based on patient flow. By focusing on private dental clinics, the research team was able to efficiently access the target population of older dental patients and collect the necessary data to examine the association between oral hygiene knowledge and practices. The data were collected between November 17, 2022, and December 18, 2022.

The source population was older dental patients treated in private dental clinics in Addis Ababa. The study population consisted of older dental patients receiving treatment in private dental clinics during the data collection period. Dental patients who were 65 years or older were included in the study, whereas older dental patients who were unable to communicate due to their health condition were excluded.

A convenience sampling method was used to select study participants. The sample size was calculated using a formula for the proportion of a single population, considering the following assumptions: p = 32%, the prevalence of oral hygiene knowledge [10], a confidence level of 95%, and a margin of error of 5%. The initial sample size was less than 10,000 people, and a finite population correction formula was used to calculate the optimal sample size. The final sample size was 111 study participants.

$${{{{{\rm{n}}}}}} 	 ={{{{{\rm{Z}}}}}}{{{{{\rm{\alpha }}}}}}/{{2}^{2}} \, {}^{\ast }{{{{{\rm{p}}}}}} {}^{\ast} (1-{{{{{\rm{p}}}}}})/{{{{{\rm{d}}}}}}2\\ 	 ={(1.96)}^{2}(0.32)(1-0.32)/{(0.05)}^{2}$$

$${{{{{\rm{n}}}}}}=334({{{{{\rm{calculated}}}}}}\; {{{{{\rm{sample}}}}}}\; {{{{{\rm{size}}}}}})+33.4(10 \% {{{{{\rm{nonresponse}}}}}}\; {{{{{\rm{rate}}}}}})$$

$${{{{{\bf{n}}}}}}={{{{{\bf{367}}}}}}$$

Since the total population was 160, the population correction formula was used to correct the size of the sample;

$${{{{{\rm{Correction}}}}}}\; {{{{{\rm{formula}}}}}}={{{{{\bf{n}}}}}}/({{{{{\bf{1}}}}}}+{{{{{\bf{n}}}}}}/{{{{{\bf{N}}}}}})\\ =367/(1+367/160)\\ ={{{{{\mathbf{111}}}}}}({{{{{\mathbf{sample}}}}}}\; {{{{{\mathbf{size}}}}}})$$

A structured questionnaire was developed based on a review of the literature. The data collection instrument included three parts: questions that evaluated sociodemographic characteristics, questions about the participant’s level of knowledge about oral hygiene, and questions about oral hygiene practices. The questionnaire was prepared in English, translated into Amharic, and back-translated to English to confirm its accuracy. The questionnaire was pretested in 5% of older dental patients attending an unselected dental clinic to test its feasibility and clarity. The questionnaire was revised and refined to improve its validity and reliability based on the pretest results. Moreover, the content and face validity of the questionnaire were measured based on the experts’ judgments.

Data collectors and supervisors were trained on the purpose of the study, questionnaire administration, and data collection procedures. An interviewer’s structured Amharic version of the questionnaire was used for data collection. Four nurses collected the data, and four dental specialists supervised the data collection procedure. The completeness and consistency of the data were checked. The coded data were entered, cleaned, and analyzed using SPSS version 23 software. Descriptive statistics were used to describe the data. Logistic regression was applied to measure the association between oral hygiene knowledge and oral hygiene practices. Multiple logistic regression was used to identify associated factors. Statistical significance was assessed at the 0.05 level. In the bivariate analysis, those variables that were statistically significant at the 0.05 level were included in the final model. All variables were considered independent variables for the outcome variable.

Ethical approval was obtained from the Research Ethics Committee of the Atlas College of Health Sciences (Ref. No: ACHS/001/22). A letter of support was provided to the Nash Specialty Dental Clinic, the Sitamin Medium Dental Clinic, the Dent-Efrata Medium Dental Clinic, and the Merit Medium Dental Clinic. Participants were informed of the purpose of the study, and informed consent was obtained before the study began. Once permission was obtained to conduct the study, each ethical procedure was strictly followed throughout the study.

### Good knowledge

Participants who knew that dental plaque and calculus were harmful believed that they would maintain oral health through brushing, using dental floss, and performing regular dental check-ups for professional cleaning; scores ranged from 7 to 9 for 9 questions about oral hygiene knowledge [20].

### Poor knowledge

A participant who is unaware that dental plaque and calculus are harmful and who does not believe in maintaining oral health through brushing, using dental floss, or performing regular dental check-ups for professional cleaning scores 0 to 6 on 9- knowledge questions [20].

### Good oral hygiene practices

Participants who brushed their teeth twice or more a day, cleaned their tongue and interdental spaces, changed their brush every 3 months, and had regular professional cleaning scores of 5 to 8 from eight oral hygiene practice questions [13].

### Poor oral hygiene practice

Participants who sometimes cleaned their teeth, never changed their brush regularly, never had professional cleaning, and never cleaned their teeth scored 0 to 4 on 8 oral hygiene practice questions [13].

## Results

### Sociodemographic characteristics of the study participants

A total of 111 participants were recruited; the mean age was 70.31 years, and the age range was 65-100 years. Of all participants, 95 (85.6%) were aged <75 years. Regarding sex, 58 (52.3%) were women, and 71 (64.0%) were married. Regarding educational status, 38 (34.2%) participants could read and write. Regarding occupation, 34 (30.6%) participants were unemployed (Table 1).

**Table 1:** Sociodemographic characteristics of the study participants in Addis Ababa, 2023 (*n* = 111).

Sociodemographic Characteristics	Category	Frequency (*N*)	Percent (%)
Age	<75	95	85.6
	>=75	16	14.4
Sex	Female	58	52.3
	Male	53	47.7
Marital Status	Single	12	10.8
	Married	71	64.0
	Widowed	17	15.3
	Divorced	11	9.9
Education Level	Illiterate	21	18.9
	Can read and write	38	34.2
	Elementary/Junior	22	19.8
	High school	10	9.0
	Higher education	20	18.0
Financial need	self- dependent	86	77.5
	depend on others	25	22.5
Self-care	Self-Dependent	105	94.6
	Dependent on others	6	5.4
Occupation	Unemployed	34	30.6
	Employed	31	27.9
	Self-Employed	22	19.8
	Business-Owner	24	21.6

### Participants’ knowledge of oral hygiene

Of these, 109 (98.2%) agreed that maintaining oral health is extremely important, and 69 (62.2%) knew about the correlation between oral health and general health. A total of 72 (64.9%) patients knew that gum bleeding was a sign of gum disease. Although most of them knew that their gum bleeding was abnormal, only 46 (41.4%) thought that the use of toothpaste and brushes was a preventive measure. Of the study participants, 50 (45.0%) correctly responded to plaque and calculus, and 107 (96.4%) mentioned that plaque and calculus were unhealthy and needed to be removed. Only 13 (11.7%) of the participants thought that their teeth should be cleaned three times a day (morning, day, and night), and 37 (33.3%) believed that cleaning twice a day (morning and night) was sufficient. Sixty-six (59.5%) participants knew that their teeth should be cleaned after meals. Sixty-three (56.8%) and 41 (36.9%) participants thought that professional tooth cleaning and routine dental checkups were obligatory, respectively (Table 2).

**Table 2:** The correct responses to the knowledge questions of the study participants in selected dental clinics, 2023 (*n* = 111).

S/N	Variables	Frequency (*N*)	Percent (%)
1	Oral health is related to general health		
	Yes	69	62.2
2	Gum bleeding indicates		
	Gum disease	72	64.9
3	Prevent gum bleeding		
	By using a toothbrush and paste	46	41.4
4	Dental plaque and calculus mean		
	Soft and hard debris that contains bacteria	50	45.0
5	Dental plaque and calculus		
	Presence is abnormal/Unhealthy/ must be removed	107	96.4
6	How often teeth must be cleaned		
	Three times a day (Morning, day and night)	13	11.7
	Twice a day (Morning and night)	37	33.3
7	Teeth must be cleaned		
	After meal	66	59.5
8	Professional cleaning is necessary		
	Yes	63	56.8
9	Routine dental check-ups are mandatory		
	Yes	41	36.9

### Participants’ oral hygiene practices

A high percentage of the participants, 106 (95.5%), indicated that they had cleaned their teeth. However, only 6 (5.4%) and 26 (23.4%) study participants cleaned their teeth three times a day and twice a day, respectively. Regarding items related to cleaning teeth, 48 (43.2%) stated that they used a toothbrush and paste, and 40 (36%) reported that it took three minutes or more to brush their teeth. Regarding cleaning of the tongue and interdental space, 28 (25.2%) and 20 (18%) participants, respectively, stated that they cleaned their tongues and interdental spaces. Most of the respondents did not use cleaning services regularly and only seven (6.3%) patients had professional cleaning every six months or less. In addition, 25 (22.5%) of respondents changed their brushes every three months (Table 3).

**Table 3:** Responses to practice questions in selected dental clinics in Addis Ababa, 2023 (*n* = 111).

S/N	Variables	Frequency (*N*)	Percent (%)
1.	**Do you brush your teeth?**		
	Yes	106	95.5
2	**How often do you clean your teeth?**		
	Three times a day	6	5.4
	Twice a day	26	23.4
3	**What do you use to clean your teeth?**		
	Toothbrush and paste	48	43.2
4	**Duration of brushing**		
	Greater than 3 minutes	40	36.0
5	**Do you clean your tongue?**		
	Yes	28	25.2
6	**Do you clean interdental space?**		
	Yes	20	18.0
7	**Last time you had professional cleaning of teeth**		
	6 months and less	7	6.3
8	**How often do you change your brush?**		
	Every three month	25	22.5

### Prevalence of oral hygiene knowledge and practices for older people

**Fig. 1:**
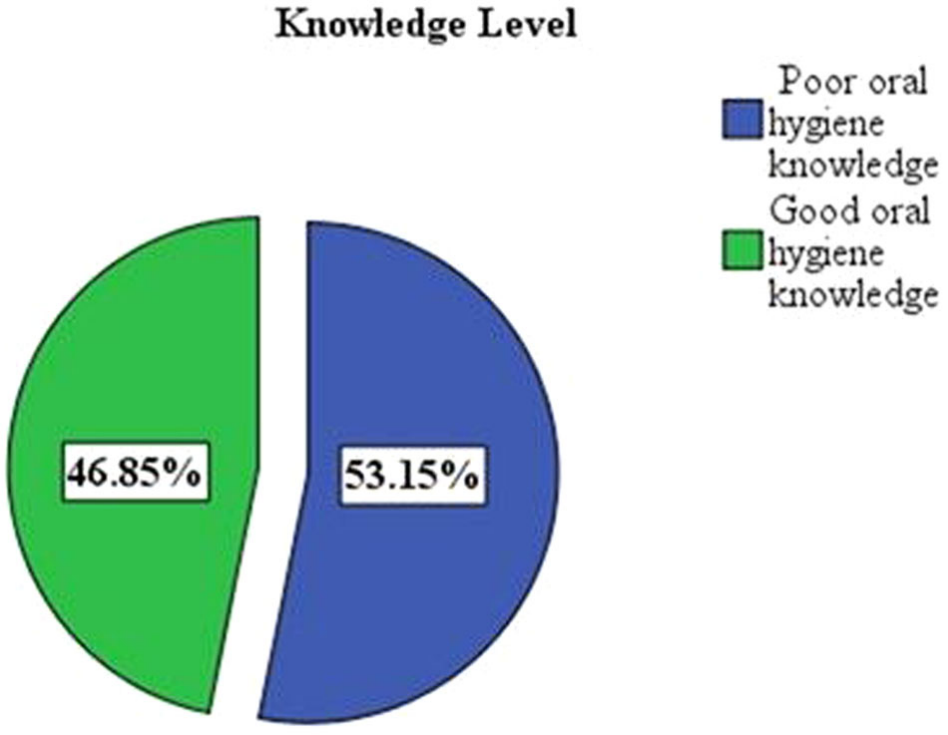
Oral hygiene knowledge of participants in selected dental clinics, Addis Ababa, 2023.

**Fig. 2:**
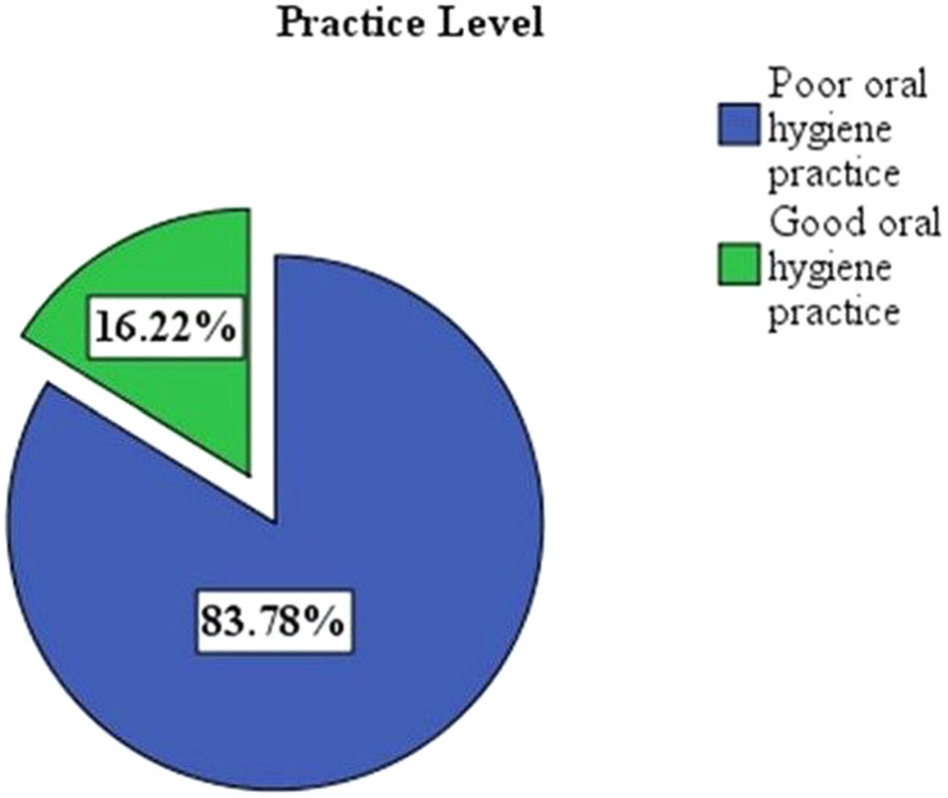
Oral hygiene practices of participants in selected dental clinics, Addis Ababa, 2023.

The prevalence of poor oral hygiene knowledge was 53.2% (Fig. 1), and the prevalence of poor oral hygiene behavior was 83.8% (Fig. 2).

### Factors associated with oral hygiene knowledge

According to the multivariate analysis, participants’ age, marital status, and education level were significantly related to their knowledge of oral hygiene. Oral hygiene knowledge was 6.2 times greater among respondents aged <75 years than among those aged 75 years. (AOR, 6.2; 95% CI, 1.13–34.2). Compared with single participants, married participants were 11.7 times more likely to have good oral hygiene knowledge (AOR, 11.7; 95% CI (1.4–94.9)). Participants with elementary or secondary education had 11.7% more oral hygiene knowledge than those who were once illiterate (AOR, 11.7; 95% CI (1.8–74.9)) (Table 4).

**Table 4:** Multivariable analysis of associations between participants’ level of knowledge and sociodemographic variables at selected dental clinics in Addis Ababa, 2023 (*n* = 111).

Variables	Category	Knowledge level		AOR (95% CI)	*P* value
		Good	Poor		
Age	<75	48 (50.5)	47 (49.5)	6.2 (1.13-34.2)	0.036
	>=75	4 (25.0)	12 (75.0)		
Sex	Female	23 (39.7)	35 (60.3)		
	Male	29 (54.7)	24 (45.3)	0.9 (0.3-2.9)	0.884
Marital Status	Single	6 (50.0)	6 (50.0)		
	Married	37 (52.1)	34 (47.9)	11.7 (1.4-94.9)	0.021
	Widowed	5 (29.4)	12 (70.6)	4.4 (0.4-42.9)	0.207
	Divorced	4 (36.4)	7 (63.6)	2.7 (0.2-40)	0.474
Education level	Illiterate	4 (19.0)	17 (81.0)		
	Can read and write	9 (23.7)	29 (76.3)	1.5 (0.3-7.0)	0.574
	Elementary	12 (54.5)	10 (45.5)	11.7 (1.8-74.9)	0.010
	High school	9 (90.0)	1 (10.0)	124.1 (5.7-2683)	0.002
	Higher education	18 (90.0)	2 (10.0)	637.3 (26.5-15314)	0.000

### Factors associated with the oral hygiene practices of respondents

According to the multivariate analysis, the respondents’ level of education and knowledge were found to be significantly associated with their oral hygiene practices (Table 5). The oral hygiene practices of participants with a higher education level were 31.1 times more likely to indicate good oral hygiene practices than those who were illiterate (AOR, 31.1; 95% CI (2.1–463.5)).

**Table 5:** Multivariable analysis of factors associated with oral hygiene practices in Addis Ababa, 2023.

Variables	Category	Practice level		AOR (95% CI)	*P* value
		Good	Poor		
Age	<75	15 (15.8)	80 (84.5)	0.3 (0.1–1.7)	0.181
	>=75	3 (18.8)	13 (81.2)		
Sex	Female	9 (15.5)	49 (84.5)		
	Male	9 (18.0)	44 (83.0)	0.6 (0.2–2.2)	0.437
Marital Status	Single	4 (33.3)	8 (66.7)		
	Married	12 (16.9)	59 (83.1)	0.5 (0.1–3)	0.451
	Widowed	1 (5.9)	16 (94.1)	0.12 (0.01–1.7)	0.120
	Divorced	1 (9.1)	10 (90.9)	0.2 (0.01–4.1)	0.307
Education level	Illiterate	1 (4.8)	20 (95.2)		
	Can read and write	2 (5.3)	36 (94.7)	0.8 (0.05–10.9)	0.849
	Elementary/ Junior	4 (18.2)	18 (81.8)	2.4 (0.2–29.8)	0.507
	High school	3 (30.0)	7 (70.0)	9.3 (0.6–153.7)	0.121
	Higher education	8 (40.0)	12 (60.0)	31.1 (2.1–463.5)	0.013
Knowledge level	Good	15 (28.8)	37 (71.2)	7.6 (2.05–27.9)	0.002
	Poor	3 (5.1)	56 (94.9)		

Participants’ oral hygiene knowledge demonstrated a significant association with their oral hygiene practices. Participants with good oral hygiene knowledge were 7.6 times more likely to follow good oral hygiene practices.

## Discussion

This study examined the association between oral hygiene knowledge and practices among 65-year-old dental patients in Addis Ababa, Ethiopia.

In this study, more than half of the participants demonstrated good knowledge on topics such as the need for professional cleaning, the importance of maintaining oral health, its relationship with general health, gum bleeding, plaque, calculus removal, and tooth hygiene after meals. In contrast, this study showed that less than half of the respondents were aware of plaque and calculus, toothbrushes, and paste as preventive measures, the frequency of brushing their teeth, and the necessity of routine dental checkups. The prevalence of poor oral hygiene knowledge was 53.15%. A study by Zhu et al. examined the knowledge and practices of Chinese adults (35–74 years old) about their oral health and revealed that knowledge about the causes and prevention of dental diseases was low [21]. Similarly, Horowitz et al. [22] demonstrated a low level of knowledge regarding the prevention of dental caries. In contrast, a study in the United Arab Emirates showed that many adult participants had high levels of oral health knowledge [23]. Similarly, the findings of a study by McQuistan et al. [24] reported that participants were aware of oral hygiene recommendations and basic dental procedures. This difference was due to differences in the age groups included in the studies.

Regarding their practices, only 16.22% of the participants had good oral hygiene practices, which is similar to the findings of the study carried out in Ibadan (SEGLA), which reported that only 12.46% of the participants were considered to have good oral hygiene practices [13].

The results of this study showed that the educational level of the participants was related to their knowledge and oral hygiene practices, which is consistent with the findings of a previous study [25] in which participants’ educational level was shown to be a significant factor for differences in health literacy. Similarly, a study in China among the Hong Kong community indicated a correlation between educational level and oral hygiene knowledge [16]. Another study also showed that educational level influences students’ knowledge of oral health. Respondents with higher education levels brushed their teeth twice a day, had clean interdental spaces, had regular dental check-ups, and used fluoride toothpaste [20].

Chen et al. [26] reported that participants with higher education levels tended to have better oral health knowledge and oral hygiene practices. However, a study conducted in the United Arab Emirates [23] revealed that education level was not associated with health knowledge. The literature is inconclusive regarding the association between educational status and oral health knowledge, which could be due to differences in the data collection instruments used between different study participants.

Age and marital status were associated with knowledge of oral hygiene. According to these findings, as age increased, a decrease in knowledge of oral care was observed among the participants of this study. Oral hygiene knowledge was greater among those aged <75 years than among those aged 75 years. However, age was not found to be a factor associated with oral hygiene practices, and this result is the same as that of Hong Kong community research in China, which reported no correlation between age and oral hygiene practices (1s7).

Marital status was associated with oral hygiene knowledge, and those who were single had lower oral hygiene knowledge than married participants. In contrast, being alone at an older age negatively impacts self-awareness, and this incorporates oral healthcare [16]. This may be related to family support, improving awareness, and encouraging the use of dental services.

Another major finding of this study was the importance of oral hygiene knowledge in hygiene practices. This study revealed that the oral hygiene knowledge of older adults was significantly associated with their oral hygiene practices. This result is consistent with a study conducted in Egypt by Abd Allah et al. [14] reported a significant positive correlation between knowledge of oral health and oral self-care practices among older people. This may be due to the impact of a higher level of awareness of oral health on good oral health practices among older patients.

### Strengths and limitations of the study

This study has several strengths, as it was conducted among older patients, who are a vulnerable and neglected portion of the population. This study has several limitations, such as its cross-sectional design and inability to establish temporal relationships among variables. This study used convenience sampling, which prevents inference among all older patients in Ethiopia. The study participants were older patients who sought dental care, which might not represent older patients who did not attend dental clinics. The calculation of sample size used a prevalence of oral hygiene knowledge derived from a study conducted in British Columbia, Canada. Given the population differences, this prevalence may not be representative of the study population in Ethiopia. The reliability of the questionnaire was not measured. Another limitation was the small sample size used. Despite the limitations mentioned above, the findings of the study can serve as a baseline for the development of health promotion interventions and other studies. Future studies will be performed to address the limitations of this study.

## Conclusions

The study revealed inadequate oral hygiene knowledge and practices among respondents. This study also demonstrated a strong correlation between knowledge and oral hygiene practices among older patients. Education level was significantly associated with oral hygiene knowledge and practices among older patients. Additionally, the age and marital status of the respondents were related to their knowledge of oral hygiene. This study showed that there is a need to expand oral health education for older people to improve their awareness and oral hygiene practices.

## Data Availability

The data used for this study are included in the article.
